# EuCAP, a Eukaryotic Community Annotation Package, and its application to the rice genome

**DOI:** 10.1186/1471-2164-8-388

**Published:** 2007-10-25

**Authors:** Françoise Thibaud-Nissen, Matthew Campbell, John P Hamilton, Wei Zhu, C Robin Buell

**Affiliations:** 1 The Institute for Genomic Research, 9712 Medical Center Dr, Rockville, MD 20850, USA; 2 J. Craig Venter Institute, 9704 Medical Center Dr, Rockville, MD 20850, USA; 3 Pioneer Hi-Bred International, 7300 NW 62^nd ^Ave, Johnston, IA 50131, USA; 4 Department of Plant Biology, Michigan State University, East Lansing, MI 48824, USA

## Abstract

**Background:**

Despite the improvements of tools for automated annotation of genome sequences, manual curation at the structural and functional level can provide an increased level of refinement to genome annotation. The Institute for Genomic Research Rice Genome Annotation (hereafter named the Osa1 Genome Annotation) is the product of an automated pipeline and, for this reason, will benefit from the input of biologists with expertise in rice and/or particular gene families. Leveraging knowledge from a dispersed community of scientists is a demonstrated way of improving a genome annotation. This requires tools that facilitate 1) the submission of gene annotation to an annotation project, 2) the review of the submitted models by project annotators, and 3) the incorporation of the submitted models in the ongoing annotation effort.

**Results:**

We have developed the Eukaryotic Community Annotation Package (EuCAP), an annotation tool, and have applied it to the rice genome. The primary level of curation by community annotators (CA) has been the annotation of gene families. Annotation can be submitted by email or through the EuCAP Web Tool. The CA models are aligned to the rice pseudomolecules and the coordinates of these alignments, along with functional annotation, are stored in the MySQL EuCAP Gene Model database. Web pages displaying the alignments of the CA models to the Osa1 Genome models are automatically generated from the EuCAP Gene Model database. The alignments are reviewed by the project annotators (PAs) in the context of experimental evidence. Upon approval by the PAs, the CA models, along with the corresponding functional annotations, are integrated into the Osa1 Genome Annotation. The CA annotations, grouped by family, are displayed on the Community Annotation pages of the project website , as well as in the Community Annotation track of the Genome Browser.

**Conclusion:**

We have applied EuCAP to rice. As of July 2007, the structural and/or functional annotation of 1,094 genes representing 57 families have been deposited and integrated into the current gene set. All of the EuCAP components are open-source, thereby allowing the implementation of EuCAP for the annotation of other genomes. EuCAP is available at .

## Background

Accurate and consistent annotation of genomes presents a challenge that can be partially solved by automated and semi-automated annotation methods. Improvements in the structural annotation of gene models can be obtained through training of *ab initio *gene finders and, for eukaryotes, through empirical transcript support in the form of Expressed Sequence Tags (ESTs) and, more critically, full-length cDNAs [1-3]. In addition to structural annotation, in large-scale genome annotation projects functional annotation is performed in an automated manner and on an individual gene basis (using sequence homology and domain searches), and would greatly benefit from manual inspection. Indeed, comparative analysis of related genes can provide important clues for the structural annotation of related proteins and assist in generating a homogeneous naming scheme at the family level.

The Institute for Genomic Research (TIGR) Rice Genome Annotation database (hereafter referred to as the Osa1 Genome Annotation database) contains the structural and functional annotation of the *Oryza sativa *L. ssp. *japonica *cv. Nipponbare genome [4]. It is the product of a mature pipeline that integrates ESTs and full-length cDNAs with the FGENESH *ab initio *gene predictions, using the Program to Assemble Spliced Alignments (PASA [2]) for structural annotation. Functional annotation is achieved through BLAST and domain searches (see details in [5,6]). Despite the availability of over 1.1 million ESTs and 33,000 full-length cDNAs [7], 28,006 of the total 51,286 non-transposable element-related gene models predicted in the rice genome have no or partial experimental support for their structure. Given the lack of experimental evidence, it is likely that some of these models are mis-annotated at the structural level. Additionally, some genes are probably undetected by our pipeline, as suggested by Massively Parallel Signature Sequence (MPSS) data [8].

Since the sequencing of the human genome, 'open annotation' or reaching out to biologists with expertise in an organism of interest or in particular gene families has been recognized as a powerful way to improve the automated annotation of sequenced genomes through manual review [9]. In addition, participation by the community helps maintain up-to-date sets of models after the end of an initial annotation project. Several approaches have been used to solicit community involvement including 1) 'jamborees' where annotation is performed by a select group of experts gathered in the same location for a short period of time as for Drosophila [10] and mouse [11], 2) organized, dispersed efforts that are limited in time as done with honeybee [12], or 3) on-going dispersed submissions from the community (Arabidopsis [13], *Caenorhabditis elegans *[14], *Pseudomonas aeruginosa *[15]).

Participants in the Rice Annotation Project (RAP) manually annotated 28,540 protein-coding genes supported by full-length cDNA from rice or other monocots [16] using the second approach. However, open-reading frames (ORFs) were assigned functions on a gene-by-gene basis, similar to our automated pipeline. In addition, only genes with EST or cDNA evidence were annotated. In Arabidopsis, several hundred genes predicted exclusively by *ab initio *prediction programs or through comparative alignment with *Brassica *have been experimentally validated [17-20], suggesting that many low-expression genes or genes with very circumscribed expression patterns may be missed by the RAP effort.

To improve the quality of the rice genome annotation, we have developed EuCAP (Eukaryotic Community Annotation Package), a portable and flexible system for 1) the submission of structural and functional annotation of genes by dispersed Community Annotators (CAs), 2) the evaluation of CA contributions by Project Annotators (PAs), and 3) the incorporation of CA contributions into our ongoing annotation effort.

Most approaches for the management of community input have aimed at "bringing annotation into the mainstream" and consists of developing tools for members of the community to annotate models *de novo *through a web interface (such as yrGATE [21], PeerGAD [22] or ASAP [23]). Although this gene-by-gene approach has its benefits, it is ill-suited for the direct integration of previous knowledge by the CA and it requires that the CA be comfortable weighing the experimental evidence presented to him/her. Like PseudoCAP, which was designed for the annotation of *P. aeruginosa *[15], EuCAP has the advantage of also accepting cDNA sequences as input. This feature allows the CAs to directly submit models annotated previously by their method of choice. In addition, the ability to input sequence makes the EuCAP pipeline coordinate-independent, and allows researchers using rice pseudomolecules (virtual contigs representing the 12 rice chromosomes [6]) built at different time or by different groups to submit their annotations without the difficulty of converting coordinates. EuCAP aims to make the submission of pre-determined gene structures as simple as possible while also allowing, if desired, the visualization and modification of these models in the context of available evidence.

**Figure 1:**
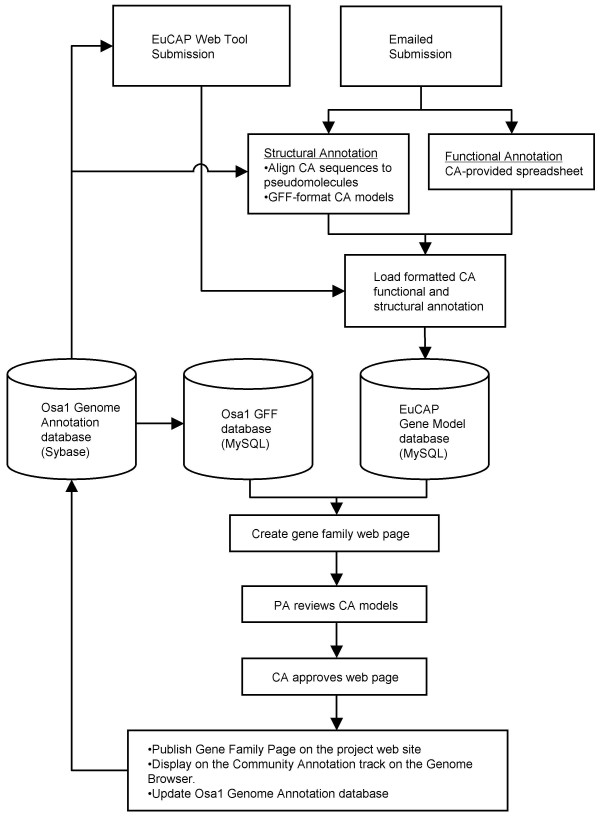
**Flow of information through the EuCAP pipeline**. Annotations are submitted through the EuCAP Web Tool or emailed, formatted, and loaded into the EuCAP Gene Model database. Using information from the Osa1 GFF database (containing feature coordinates of Osa1 models) and the EuCAP Gene Model database, a customized web page is generated for each family. After review of the model by the PA and approval of the pages by the CA, the web pages are made public, the CA models are added to the Genome Browser and used to update the Osa1 Genome Annotation database.

The EuCAP pipeline relies on two MySQL databases. The EuCAP Gene Model database is populated with structural and functional annotations provided through the EuCAP Web Tool or via email to a PA. The Osa1 GFF database contains the Generic Feature Format (GFF)-formatted models of the Osa1 Genome Annotation (Figure 1). Customized Perl scripts facilitate the population of these databases and the generation of web pages displaying the CA model and any overlapping models from the latest release of the Osa1 Genome Annotation. Upon acceptance by the PA, the CA model is integrated into the Osa1 Genome Annotation [24]. As of July 2007, over 1,000 models have been contributed and approved for incorporation in the Osa1 gene set.

## Implementation

### EuCAP Gene Model database

The EuCAP Gene Model database is a MySQL database that stores the functional and structural annotation submitted by the CAs. The schema consists of six tables: Users (CA information), Family (gene family information), Superfamily (optional information linking gene families), Loci (submitted functional annotation), Structural_annotation (submitted structural annotation stored in GFF), and Sessions (a utility table for CGI session management). A file with the SQL commands needed to set up the database tables is included in the package available on sourceforge or in additional file [Supplementary-material S1].

### Osa1 GFF database

The Osa1 GFF database uses the BioPerl Bio::DB::GFF schema and is loaded with GFF-formatted annotation from the current version of the Osa1 Genome Annotation database [24]. It is used by the EuCAP Web Tool and the web page generation scripts described below. It is also the backend for the Osa1 Genome Browser.

### EuCAP Web Tool

The Web Tool, available for testing at [25], is composed of a server side Perl CGI script, the EuCAP Gene Model database, and client side Javascript. The web pages are stored as templates and can therefore be customized and integrated into the style of an existing website. They are dynamically completed using HTML::Template. The Class::DBI module is used to provide an Object Relational Mapper (ORM) layer for database access. Session management is provided by the CGI::Session module. User authentication is provided by the Authen::Passphrase modules. Several BioPerl modules are used to access the Osa1 GFF and EuCAP Gene Model databases, generate images, perform BLAST searches, and parse BLAST output.

### Handling of email submissions

Input for the EuCAP pipeline consists of a cDNA sequence for each CA model and of an Excel spreadsheet containing functional information for the CA model(s). The first step of the pipeline is to determine the Osa1 locus identifiers (LOC_OsXXgXXXX) with which the CA models overlap. This is done by searching the cDNAs against the rice pseudomolecules using GMAP (the Genomic Mapping and Alignment Program, [26]). A Perl script parses the GMAP compressed output and searches the mapped coordinates against the Osa1 Genome Annotation Database to identify the Osa1 models overlapping with the CA models. The spreadsheet, complete with the Osa1 locus identifiers or mapping coordinates if no Osa1 model is defined at that locus, are loaded into the Loci table of the EuCAP Gene Model database. The cDNAs of the CA models are aligned to the rice pseudomolecule twice using GMAP with different set of options. Output of the -S -n 1 -T options contains alignments truncated to the largest open reading frame and, by comparison to the full alignment output (-S -n 1 options), allows the calculation of the untranslated regions (UTRs). The two alignments are parsed and a GFF output of the models' structures is created. The GFF file is loaded into the Structural_annotation table of the EuCAP Gene Model database. The Superfamily, Family, and Users table of the EuCAP Gene Model database are populated based on information provided by the CA in the spreadsheet.

### Display of the Community Annotation

Once the functional and structural annotation for a gene family is loaded into the EuCAP Gene Model database, Perl scripts generate the web pages using a HTML template and the BioPerl Bio::Graphics interface to create the images of the Osa1 and CA models.

### Incorporation of the CA models to a new release of the Osa1 Genome Annotation

CA models are first evaluated against the most recent release of the Osa1 Genome Annotation. When a new release is launched, the models predicted by our automated pipeline are substituted with the adopted CA models. In the fall of 2006, our automated annotation pipeline was run in preparation for Release 5 of our annotation. Perl scripts were used to compare the CA models to the new gene predictions based on their GFF-formatted coordinate information. In cases where the Osa1 model differed from an accepted CA model, the coordinates of the Osa1 model were adjusted in the Osa1 Genome Annotation database to the CA model using Neomorphic's Annotation Station [27]. Models missed by the automated pipeline were also incorporated in the final release through Annotation Station. The CA functional annotations (in the form "gene name-gene description") were used to overwrite the automated annotation in the Osa1 Genome Annotation database. Afterward, a copy of the EuCAP Gene Model database was made. The locus identifiers and the functional annotation of the locus linked to the CA models were changed in the new database when different from the previous release. This new database was used to make CA web pages reflecting the changes in the Osa1 Genome Annotation.

## Results and Discussion

### The submission and curation process

Annotations can currently be submitted to the project in two ways to provide maximal ease and participation of CAs. The EuCAP Web Tool was constructed for the CA to contribute structural and functional annotation of genes and gene models via a web-based portal [25,28]. In the first page of the EuCAP Web Tool, the user is prompted to enter a model or locus identifier, or alternatively, perform a BLAST search against the rice pseudomolecules using a protein or nucleotide sequence to identify the gene or locus to be annotated. Once the corresponding Osa1 model or the locus is found, the CA provides the name and description for the gene. Additional functional annotation such as source (publication, website, etc...), criteria used to determine the function, CA comments, putative subcellular localization, primer sequences, accession numbers of supporting evidence, or mutant lines can also be entered.

**Figure 2:**
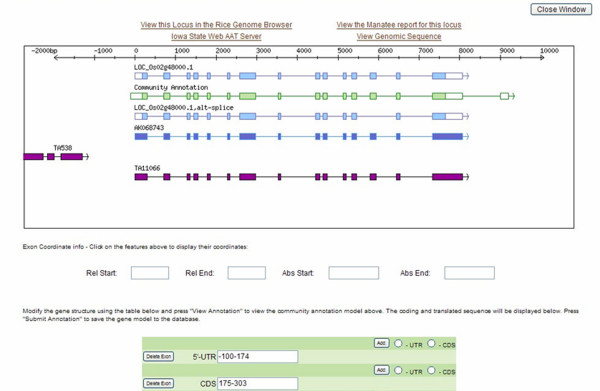
**Online form for the submission of structural annotation through the EuCAP Web Tool**. The Osa1 annotation (light blue), the submitted model (green), full-length cDNAs (dark blue) and rice Transcript Assemblies (purple) are shown in the viewer. Coordinates of the features can be displayed by mousing over the features. Exon and exon coordinates of the CA model can be modified in the fields at the bottom of the page

Structural annotation can also be performed by the CA (Figure 2). The submitted model can be visualized alongside the Osa1 model(s), Rice Transcript Assemblies (which are clustered assemblies of ESTs, full length cDNAs, and mRNAs [29]) as well as full-length cDNAs aligning in the same region. In this interactive page, coordinates of the exon features of the depicted evidence and models are available through hyperlinks. New exons, updated coordinates and UTRs can be added to the CA model, and viewed in the light of the available evidence. Links to the Osa1 Rice Genome Browser [6], the Manatee page in which structural and functional evidence such as protein domains and BLASTP search results are collated [30], the Iowa State Web AAT server [31], and the genomic sequence are provided to assist the CAs in refining their annotation. Upon submission, the coordinates of the contributed model are stored in the EuCAP Gene Model database.

While on-line submission is well-suited for a small number of genes, or in cases where the CA would like to edit online his/her models based on the current Osa1 model or experimental evidence, it is tedious for large families and redundant when the gene model structure has already been established by the CA. Therefore, we have developed an e-mail-based submission process thereby providing CAs the option of emailing the models' cDNA sequences (or just the Osa1 model identifiers if the CA models are identical to current or previous Osa1 models) along with a preformatted Excel spreadsheet containing the gene names and descriptions, a brief description of the annotation methods used, and supporting cDNA or protein accessions. These data are then readily used to populate the Loci and Family tables of the EuCAP Gene Model database using a simple script.

### Automated display of alignment of the CA model

**Figure 3:**
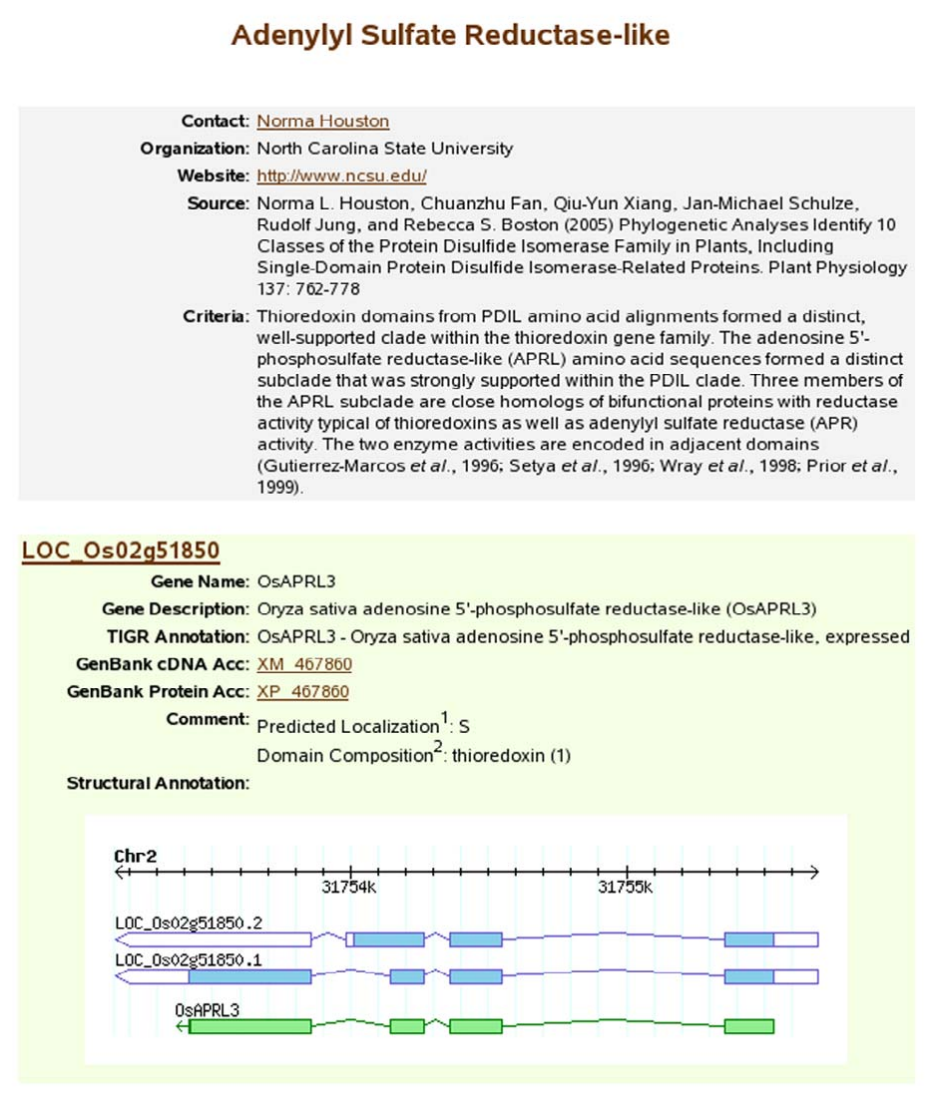
**An example of a Community Annotation web page**. The page, titled after the family annotated (adenylyl sulfate reductase) contains the contact information of the CA, a reference for the annotation, and a summary of the method used for the annotation. For each CA model, the locus is linked to the Rice Genome Browser and the alignment of the CA (in green) and the Osa1 models (in blue) is shown. Gene name and function provided by the CA, accession(s) for the genomic, cDNA and/or protein sequence(s) and other information of interest are listed and hyperlinked to further web pages.

Alignment of the CA-provided cDNA sequences onto the rice pseudomolecules and prediction of the ORFs in the cDNA are performed using GMAP [26]. The alignments are converted to GFF and loaded into the Structural_annotation table of the the EuCAP Gene Model database. Graphical views of the CA models and Osa1 models (if annotated) are generated through a Perl script that retrieves the coordinate information of the CA and of the Osa1 model with which it overlaps, from the EuCAP Gene Model and the Osa1 GFF databases, respectively. A web page featuring these graphical representations and all the information relevant to the CA models and the family (i.e. name of the annotator, criteria used, etc...) is then automatically generated (Figure 3). Hyperlinks of each locus to the Osa1 Genome Browser allow the visualization of the experimental evidence aligning at that genomic location.

### Review by Project Annotators

Prior to the public release of the community annotation, the CA-generated structural and functional annotation are reviewed by a PA. This review has three goals: 1) to ascertain that the intellectual contribution of the annotator is represented as intended in the CA webpage, 2) to communicate differences between the CA and the Osa1 models to the CA, and 3) to decide whether the CA model should be adopted and integrated into the Osa1 Genome Annotation or whether it should be rejected.

If differences exist between the structure of the CA model and that of the Osa1 model, all supporting evidence is evaluated and weighed to determine the best structural annotation of the locus, and whether the Osa1 model should be modified according to the CA. Accepted guidelines for manual curation are followed for this evaluation, namely a full-length cDNA is more trustworthy than an EST which itself is more trustworthy than a protein alignment followed by a gene prediction [32]. When no evidence is available, or when the evidence is contradictory, the CA model is favored. The final decision regarding the structure of the Osa1 model resides with the PA and is shared with the CA. However, the CA is free to represent his/her model within the CA gene family web page regardless of whether the model will be incorporated into the Osa1 Genome Annotation. The gene name and description given by the CA is adopted for the Osa1 model unless gross structural differences exist between the two models.

### Integration of the CA annotation into the Osa1 Genome Annotation

**Figure 4:**
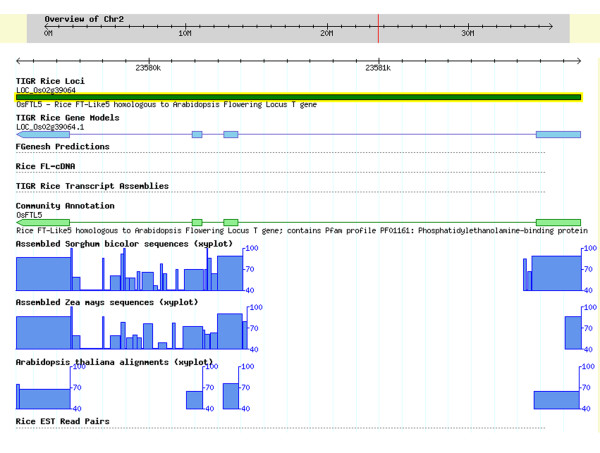
**An example of a gene "missed" by the automated annotation pipeline**. The current annotation is represented in the TIGR Rice Gene Model track. Due to lack of either a FGENESH prediction, or an EST or FL-cDNA alignment, no model was annotated prior to integration of the CA model (shown in the Community Annotation track). The xy-plot in the sorghum, maize and Arabidopsis tracks represent the percent sequence homology with rice. The CA model is supported by sequence homology of exons in Arabidopsis, maize, and sorghum.

**Figure 5:**
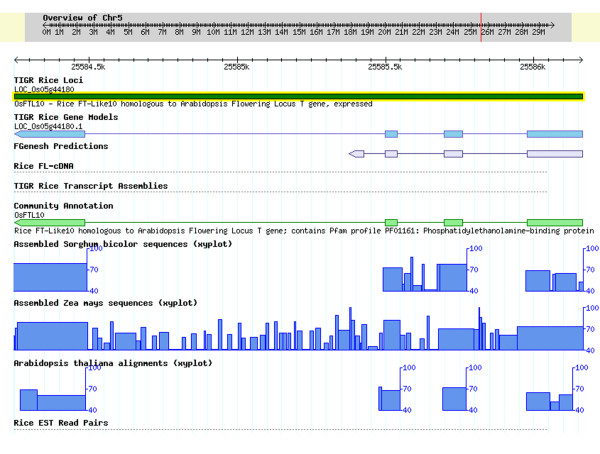
**An example of a gene misannotated by the automated annotation pipeline**. The current annotation is represented in the TIGR Rice Gene Model track. This model integrates the CA model shown in the Community Annotation track and replaces the previous (release 4) annotation corresponding to the FGENESH prediction. The xy-plot in the sorghum, maize and Arabidopsis tracks represent the percent sequence homology with rice. The CA model is supported by sequence homology of exons in Arabidopsis, maize and sorghum. Note the absence of EST or full-length cDNA evidence at this locus.

As of July 2007, 1,114 genes contributed by 18 CAs were evaluated against the Release 5 models predicted by our pipeline for incorporation into the Osa1 Genome Annotation. A total of 1,094 CA models were adopted. The structural and/or functional annotation of the 457 genes reviewed and adopted before January 2007 was incorporated into Release 5, while that of the 637 genes reviewed later will be integrated in the next release. Altogether, we found that 885 or 79% of the genes contributed were structurally identical to the Osa1 models (notwithstanding the UTRs) (Table 1), indicating the high quality of our automated pipeline. A total of 64 (5.7%) CA genes, with no corresponding locus identifiers, were "missed" by our annotation pipeline and were added to our set of models (see an example in Figure 4). In addition, 78 (7%) models had their internal structure modified (see an example in Figure 5), and 12 alternative splice forms were added (Table 1) according to the CA models. Twenty (1.8%) contributed models that either contradicted available evidence, or were most likely the product of truncated cDNAs, or contained non-consensus splice sites, were not adopted.

**Table 1:** Models deposited by Community Annotators and incorporated into the Osa1 Genome Annotation

CA models identical to Osa1 models	**885**
Functional genes	868
Putative pseudogenes	17
CA models different from Osa1 models and incorporated in the Osa1 Genome Annotation	**209**
New genes	64
Alternative splice forms	12
Improved models	78
Putative pseudogenes	55
CA models not incorporated in the Osa1 Genome Annotation	**20**
Total number of contributed models	**1,114**

A total of 72 (6.5%) of the models were annotated as pseudogenes by the CAs. These genes are thought to be non-functional due to the presence of premature stop codon(s) interrupting the reading frame, and are currently not annotated as such by our automated annotation pipeline. Seventeen of the 72 CA pseudogenes were annotated as genes by our pipeline, while 25 overlapped with annotated genes but had different boundaries or internal structure. Thirty were not annotated. Pseudogenes are difficult to identify in an automated manner. They are best recognized by comparison to functional members of the family which allows the detection of frameshifts and premature truncation. The CA platform is therefore ideal for their identification.

The most important contribution of the community effort has been toward functional annotation. Through annotation of entire families, CAs provide a nomenclature that is uniform at the family level and which is current with the published literature. We updated the functional annotation of 1,094 loci from the Osa1 Genome Annotation from the annotation assigned through our automated pipeline with the functional annotation provided by the CA.

To reflect the convergence of the community and the Osa1 models, the CA web pages and the Osa1 Genome Browser CA track were updated in January 2007 with the final Release 5 locus identifiers, model structures, and functional annotations. These overall changes were possible by the storage of the CA annotation in a dedicated database. We anticipate conducting a similar integration effort with models contributed in 2007 in the next release of the Osa1 Genome Annotation.

### Use of community annotation for the improvement of the automated pipeline

In order to identify areas for improvement for our automated pipeline, we examined cases of "missed" or wrong predictions by our pipeline in comparison to models provided by the CAs. Occurrences of missed genes resulted almost exclusively from the absence of FGENESH models, while misannotated genes resulted from inaccurate FGENESH prediction with no or partial EST support. At these loci, CA models were supported primarily by protein alignments from Arabidopsis or by aligning assemblies from maize or sorghum (Figures 4 and 5). This reflects the fact that most CA models were identified by homology to fully-characterized proteins in rice or other plants, or by phylogenetic analysis [33-35]. The incorporation of protein evidence into the automated prediction of ORF could improve the final model in such cases. To that end, we are currently evaluating the performance of Evidence Modeler [36], an algorithm incorporating *ab initio *gene predictions, cDNA and protein evidence into the gene model.

## Conclusion

We have designed EuCAP, a package for the incorporation of structural and functional annotation from the community into an on-going annotation effort. Sets of simple Perl scripts allow the PAs to quickly enter the information in a database, integrate the CA annotation in the current project annotation and generate web pages. These pages represent the community models, credit the CAs, and provide a centralized repository for their annotation.

EuCAP was designed to be portable and is freely available to other community annotation projects (see Availability and Requirements), although some modifications for local configurations may be necessary. The web pages are dynamically generated using templates and can therefore be integrated into an existing web site.

During the course of designing and validating the pipeline, we have learned that shifting the burden of the data processing from the CAs to a few trained PAs with a set of custom scripts at their disposal greatly enhances the efficiency of the submission process and stimulates greater CA participation. CAs do not have to abide by strict data format constraints but are able to simply provide sequence files. For this reason, most CAs have chosen to email their data rather than submit it with the EuCAP Web Tool.

In our experience, soliciting contributions is essential to a dynamic community annotation project. Direct requests to the authors of relevant publications, presentation of the project at meetings and announcements on our website generated the majority of the contributions. In just one year, we have successfully obtained over 1,114 manually curated genes (57 families) from members of the rice and plant community at large, incorporated this annotation into our annual update, and displayed all of the CA annotation on individualized web pages. By comparison, The Arabidopsis Information Resource lists 8,331 genes in 996 families annotated by the community [37]. However, it should be taken into consideration that the Arabidopsis genome was completed in 2000 [38], five years before the rice genome [39] and that the Arabidopsis community is larger than the rice community. To avoid the waning of interest and participation observed by other community annotation projects after their initial efforts, we are committed to maintaining a proactive strategy to solicit scientists that have made their data public to participate in the community annotation process. We expect that the full sequence of additional plant genomes, in particular maize and sorghum [40], will stimulate the use of phylogenomics approaches for the annotation of gene families in the Grasses and thereby provide more high-quality annotations to incorporate into our community annotation project.

## Availability and Requirements

• **Project name**: EuCAP

• **Project home page**: 

• **Operating system(s)**: Platform independent

• **Programming language**: Perl, Javascript

• **Other requirements**: Perl, Apache, MySQL, BioPerl 1.5.1 or higher, Perl modules: Authen::Passphrase::MD5Crypt, CGI::Session, HTML::Template, Class::DBI, DBI, DBD::mysql, IO::String, JSON

• **License**: BSD

• **Any restrictions to use by non-academics**: None

## Competing interests

The author(s) declares that there are no competing interests.

## Authors' contributions

MAC and FT reviewed the annotations, integrated them into the Osa1 Genome Annotation and helped draft the manuscript. MAC solicited the contributions. JPH designed the database wrote the pipeline and helped draft the manuscript. WZ participated in the initial design and coding portions of the prototype. CRB helped draft the manuscript. All authors read and approved the final manuscript.

## Supplementary Material

Additional File 1 Compressed folder of files necessary to install and use EuCAP. Click here for file
