# Supplementary material for: EuCAP, a Eukaryotic Community Annotation Package, and its application to the rice genome
Source: BMC Genomics. 2007 Oct 25;8:388. doi: 10.1186/1471-2164-8-388 (PMC2151081; doi:10.1186/1471-2164-8-388)
Supplement: Additional File 1 — Compressed folder of files necessary to install and use EuCAP. [file 1471-2164-8-388-S1.zip › eucap/bin/gene_family_page_template.tmpl]

TIGR Rice Community Annotation - <TMPL\_VAR NAME="gene\_family">


|  |  |
| --- | --- |
|  |  |
|  |  |
| --- | --- |
| Superfamily: |  |
| Contact: | " > |
|  |  |
| --- | --- |
| Organization: |  |
|  |  |
| --- | --- |
| Website: | "> |
| Source: |  |
| Criteria: |  |
|  |  |
| --- | --- |
|  |  |
|"> &label=Contig-Genes-Rice\_Annotation-Community\_Annotation"> | |
| Gene Name: |  |
|  |  |
| --- | --- |
| Alternate Gene Name: |  |
|  |  |
| --- | --- |
| Gene Description: |  |
|  |  |
| --- | --- |
| TIGR Annotation: |  |
|  |  |
| --- | --- |
| GenBank Genomic Acc: |  |
|  |  |
| --- | --- |
| GenBank cDNA Acc: |  |
|  |  |
| --- | --- |
| GenBank Protein Acc: |  |
|  |  |
| --- | --- |
| Mutant Line/Info: |  |
|  |  |
| --- | --- |
| Comment: |  |
|  |  |
| --- | --- |
| Structural Annotation: |  |
| " > | |
|  |  |
| --- | --- |
|  | |
|  | |
|  |  |
| --- | --- |
|  | |
