# Supplementary material for: EuCAP, a Eukaryotic Community Annotation Package, and its application to the rice genome
Source: BMC Genomics. 2007 Oct 25;8:388. doi: 10.1186/1471-2164-8-388 (PMC2151081; doi:10.1186/1471-2164-8-388)
Supplement: Additional File 1 — Compressed folder of files necessary to install and use EuCAP. [file 1471-2164-8-388-S1.zip › eucap/tmpl/annotate.tmpl]

EuCAP - Annotate Gene Family


# Community Annotation for the Gene Family

## Description:

### Add gene family members:

Please input locus identifiers:

|  |  |
| --- | --- |
|  |  |
|  |

Alternatively, please input protein OR nucleotide sequences
(in FASTA format) or locus identifiers to identify rice homologs:

Please select the type of sequences entered:

|  |  |  |  |
| --- | --- | --- | --- |
| Protein: |  | Nucleotide |  |

E value: 
1e-50
1e-20
1e-10
1e-5

---

hits for query:

| Add | Locus Hit | Length | Score | E-value | Description |
| --- | --- | --- | --- | --- | --- |
|  |  |  |  |  |  |  |
| --- | --- | --- | --- | --- | --- | --- |
| "> |  |  |  |  |  |  |

|  |  |  |  |
| --- | --- | --- | --- |
|  |  |  |  |

---

## Gene Family Members and Annotation:

|  |  |  |  |  |  |  |
| --- | --- | --- | --- | --- | --- | --- |
| |  |  |  |  | | |

|  |  |  |  |  |  |  |
| --- | --- | --- | --- | --- | --- | --- |
| Delete? | Locus | Current Annotation | Gene Name | Alt Gene Name | Gene Description | |
| "> | "> |  | " value=""> | " value=""> | " value=""> | |
| Genomic Acc | cDNA Acc | Protein Acc | Mutant Info | Comment | Struct Anno? | Open Struct Anno Page |
| " value=""> | " value=""> | " value=""> | " value=""> | " value=""> | YesNo | ')"> |


  
  

## No loci selected for this gene family.


---

|  |  |  |  |  |  |  |
| --- | --- | --- | --- | --- | --- | --- |
| |  |  |  |  | | |
