# Supplementary material for: EuCAP, a Eukaryotic Community Annotation Package, and its application to the rice genome
Source: BMC Genomics. 2007 Oct 25;8:388. doi: 10.1186/1471-2164-8-388 (PMC2151081; doi:10.1186/1471-2164-8-388)
Supplement: Additional File 1 — Compressed folder of files necessary to install and use EuCAP. [file 1471-2164-8-388-S1.zip › eucap/tmpl/final_submit.tmpl]

Submit Annotation


## Submit Annotation for

The data has been successfully submitted to the Community
Annotation Curators. Please log out and a member Community
Annotation team will contact you and keep you informed of the
progress of you submission.
