# Supplementary material for: EuCAP, a Eukaryotic Community Annotation Package, and its application to the rice genome
Source: BMC Genomics. 2007 Oct 25;8:388. doi: 10.1186/1471-2164-8-388 (PMC2151081; doi:10.1186/1471-2164-8-388)
Supplement: Additional File 1 — Compressed folder of files necessary to install and use EuCAP. [file 1471-2164-8-388-S1.zip › eucap/tmpl/login.tmpl]

EuCAP - Login


# Eucaryotic Community Annotation Package (EuCAP)

## Community Annotation Web Tool

Please log in:

### 

| Username: |  |
| Password: |  |
|  | |
|  |  |
