# Supplementary material for: EuCAP, a Eukaryotic Community Annotation Package, and its application to the rice genome
Source: BMC Genomics. 2007 Oct 25;8:388. doi: 10.1186/1471-2164-8-388 (PMC2151081; doi:10.1186/1471-2164-8-388)
Supplement: Additional File 1 — Compressed folder of files necessary to install and use EuCAP. [file 1471-2164-8-388-S1.zip › eucap/tmpl/review_annotation.tmpl]

Review Annotation


## Review of Community Annotation For: Gene Family

| Locus | Orig Annotation | Gene Name | Alt Gene Name | Gene Desc | Genomic GB Acc | cDNA GB Acc | Protein GB Acc | Mutant Info | Comment | Has Struct Anno? |
| --- | --- | --- | --- | --- | --- | --- | --- | --- | --- | --- |
|  |  |  |  |  |  |  |  |  |  |  |
| --- | --- | --- | --- | --- | --- | --- | --- | --- | --- | --- |
|  |  |  |  |  |  |  |  |  |  | YesNo |
