# Supplementary material for: EuCAP, a Eukaryotic Community Annotation Package, and its application to the rice genome
Source: BMC Genomics. 2007 Oct 25;8:388. doi: 10.1186/1471-2164-8-388 (PMC2151081; doi:10.1186/1471-2164-8-388)
Supplement: Additional File 1 — Compressed folder of files necessary to install and use EuCAP. [file 1471-2164-8-388-S1.zip › eucap/tmpl/select_family.tmpl]

EuCAP - Select Gene Family


## Select a Gene Family to Annotate

Welcome

---

User Details:

- Organization:
- Email:
- Website:

---


Gene Families

Please select the gene family you wish to annotate:

|  | Gene Family | Description |
| --- | --- | --- |
|  |  |  |
| --- | --- | --- |
| "> |  |  |

---

|  |  |
| --- | --- |
|  |  |
