# Supplementary material for: EuCAP, a Eukaryotic Community Annotation Package, and its application to the rice genome
Source: BMC Genomics. 2007 Oct 25;8:388. doi: 10.1186/1471-2164-8-388 (PMC2151081; doi:10.1186/1471-2164-8-388)
Supplement: Additional File 1 — Compressed folder of files necessary to install and use EuCAP. [file 1471-2164-8-388-S1.zip › eucap/tmpl/structural_annotation.tmpl]

Strucutral Annotation


## Structural Annotation for Locus:

Useful links for structural annotation -   
Iowa
State AAT server

" usemap="#">

Exon Coordinate info - Roll the mouse cursor over the
exon features above to display their coordinates:

Rel Start: |  | Rel End: |  | Abs Start: |  | Abs End: |  |

">
">
">
">
">
">

Modify the gene structure using the table below and press "View
Annotation" to view the community annotation model above. The coding
and translated sequence will be displayed below. Press "Submit
Annotation" to save the gene model to the database.

| Delete Feature | Feat Type | Start | Stop |
| --- | --- | --- | --- |
|  |  |  |  |
| --- | --- | --- | --- |
|  | | | |
|  | selected="selected" >CDS selected="selected">UTR | "> | "> |
|  | | | |

|  |  |
| --- | --- |
|  |  |
