# Supplementary material for: EuCAP, a Eukaryotic Community Annotation Package, and its application to the rice genome
Source: BMC Genomics. 2007 Oct 25;8:388. doi: 10.1186/1471-2164-8-388 (PMC2151081; doi:10.1186/1471-2164-8-388)
Supplement: Additional File 1 — Compressed folder of files necessary to install and use EuCAP. [file 1471-2164-8-388-S1.zip › eucap/tmpl/submit_annotation.tmpl]

Submit Annotation


## Submit Annotation for

Do you want to submit your community annotation for this gene
family to the community annotation curators and make you annotation
public on our website and databases?

|  |  |  |  |
| --- | --- | --- | --- |
| Yes: |  | No: |  |
|  | | | |
